# Determining Patients’ Satisfaction Level With Hospital Emergency Rooms in Iran: A Meta-Analysis

**DOI:** 10.5539/gjhs.v7n4p260

**Published:** 2014-01-14

**Authors:** Vida Kardanmoghadam, Nima Movahednia, Mahtab Movahednia, Mahmood Nekoei-Moghadam, Mohammadreza Amiresmaili, Mahmood Moosazadeh, Hossein Kardanmoghaddam

**Affiliations:** 1 Research Center for Health Services Administration, Institute of Future Studies in Health, Kerman University of Medical Sciences, Kerman, Iran; 2 Zabol University of Medical Sciences, Zabol, Iran; 3 Health Sciences Research Center, School of Health, Mazandaran University of Medical Sciences, Sari, Iran; 4 Faculty Member of Birjand University of Technology, Birjand, Iran

**Keywords:** patients’ satisfaction, emergency, hospital, meta-analysis

## Abstract

Emergency department is one of the important parts of hospitals, and patients’ satisfaction with this department significantly affects their overall satisfaction with the hospital. Therefore, evaluating patients’ satisfaction level with the emergency part has been taken into account in different studies. The purpose of this study was to systematically review all available primary studies and their results and to evaluate patients’ satisfaction level with emergency rooms of hospitals. In this study, previous documents were reviewed; to do this, national and international databases were searched electronically and related articles were extracted. Reference list of the published studies were also reviewed to increase sensitivity and to select a greater number of articles. Reviewing and studying titles and texts of the articles, repeated and unrelated cases were excluded. The remaining articles were entered into stat aver., 11 for meta-analysis. After meta-analysis, 24 articles were selected. The lowest and highest satisfaction level was 24 and 98.4% respectively. Meta-analysis results of studies showed that general evaluation of patients’ satisfaction level with emergency rooms of hospitals was 68.9% in Iran. This meta-analysis revealed that patients’ satisfaction level with performance and with the way services were presented in emergency rooms of hospitals was desirable in Iran. Concerning multifactorial nature of patients’ satisfaction, it is necessary to take this matter into regular and routine consideration.

## 1. Introduction

Emergency department is one of the important hospital departments whose performance affects performance of other parts of the hospital (Yekkeh, 2006). Emergency centers, as a part of a system presenting health services in the society, play an important role in satisfaction of health service consumers (Moosazadeh, Nekoei-moghadam, & Amiresmaili, 2013); that is; satisfaction level can improve by improving quality and by meeting expectations and needs of service receivers (Gilleard & Reed, 1998).

Satisfaction is the basis for selecting the emergency department by the patient or recommending a certain emergency department to other patients (Lau, 2000). Satisfaction with emergency department increases total satisfaction with the organizations that provide medical services (Carter, Pouch, & Larson, 2013). In recent decades, the number of people referring to emergency centers has increased; it is bad for patients’ safety and affects quality of care (Gilleard, & Reed, 1998; Ivy et al., 2013). Moreover, being aware of patients’ perception of medical needs and emergencies helps emergency nurses evaluate and classify patients (Ekwall, 2013). Patients and people’s viewpoints must be taken into account in order to improve quality of services (Amiresmaili & Moosazadeh, 2013).

In studies conducted in different countries, evaluating patient’s satisfaction has been emphasized in order to improve the quality of services (Moosazadeh, Nekoei-moghadam, & Amiresmaili, 2013; Gilleard & Reed, 1998; Lau, 2000; Carter, Pouch, & Larson, 2013; Ivy et al., 2013). In a study conducted at a hospital in Virginia, 87.9% of patients were satisfied with services they had received; he also stated that gender, place of living, education level, marital status, age and income were the factors that affected patient’s satisfaction (Mackinley & Roberts, 2001). In another study carried out in Kentucky, America on satisfaction level of 1056 patients with nursing attentions, it was shown that 84% were satisfied with nursing services (Trout, Magnusson, & Hedges, 2000).

In his study in Qazvin in 2000, Shaikhi and Javadi reported that patients’ satisfaction level with admission in emergency department was 94.4% (Shaikhi & Javadi, 2005). It was also 86.5% in a study carried out by Ansari et al at Iran University of Medical Science (Ansari, Ebadi, & Molasadeghi, 2005). In another study in 2007, satisfaction level with services was 72.9% (Moshiri, Nourbakhsh, Ghafari, & Shafie, 2011).

Initial electronic search revealed that different studies have been carried out concerning patients’ satisfaction with services at clinics and emergency centers of hospitals; combining findings of these studies, precious results will be extracted which will be a valuable criterion for policymakers and programmers due to their high reliability. Thus, authors tried to review all available studies systematically and to combine them using meta-analysis so as to evaluate patients’ satisfaction with emergency departments of Iran with regard to considerations related to the heterogeneity of studies.

## 2. Methods

The aim of the present study was to determine patients’ satisfaction level with emergency departments of hospitals in Iran; to do this, previous documents were reviewed.

### 2.1 Search Strategy

In this research, to find studies published electronically until December 2013, articles published in domestic and foreign journals as well as those available in Persian data bases of SID (www.sid.ir), Iranmedex (www.iranmedex.com), Magiran (www.magiran.com), Medlib (www.medlib.ir) and English database of PubMed and Google Scholar were used. In this search, Persian and English keywords and probable combination of main and important words were searched. These search was done with keywords of “Satisfaction, Emergency, hospital, Patient, Iran/Iranian and plus names of provinces and conjunctions “and/or”. The Persian Keywords were equivalent to their English counterparts and all probable combinations were considered. This search was carried out in December 2013. Also, reference list of published studies was evaluated to increase sensitivity and to select more studies. Search evaluation was done randomly by an independent researcher and it was confirmed that no studies were excluded.

### 2.2 Studies Selection

Entire text or summary of all searched articles, documents and reports were extracted. After reviewing and studying titles of documents, the repeated items were excluded; then, texts of articles were carefully studied by researchers and the related articles were selected and irrelevant ones were excluded.

### 2.3 Quality Evaluation

Having determined the related studies in terms of titles and contents, a checklist which was used in previous study (Moosazadeh, Nekoei-moghadam, Emrani, & Amiresmaili, 2014), was applied to evaluate the quality of documents; objective of every research, study method, sample size, sampling method, data collection tool, variables evaluation status and analysis status were examined using 12 questions (one score for each question) (Table 2).

**Table 1:** Patient satisfaction percent with Iranian hospitals emergency departments according to studies entered to meta-analysis and pooled estimation

ID	First author	publication year	Location of study	Average or range of age	Sample size			Satisfaction level

					Male	Female	Total	
1	Omidvari	2008	Tehran	47.3±16.39	84	69	153	41.8
2	Sarchami	2000	Ghazvin	NA*	343	650	993	98.4
3	Ansari	2004	Tehran	1-75	37	365	402	80.7
4	Ebrahimnia	2007	Tehran	NA*	227	132	360	82.4
5	Khashjan	2005	Tehran	NA*	NA*	NA*	759	80.7
6	Dirkavand	2010	Ilam	NA*	NA*	NA*	100	78
7	Peyrovi	2009	Tehran	45.32±16.06	NA*	NA*	20	67.4
8	Zahmatkesh	2006	Golestan	35.5±15.6	NA*	NA*	2400	24
9	Roodbari	2008	Zahedan	NA*	NA*	NA*	300	80.5
19	Entezari Asl	2000	Ardabil	NA*	60	40	600	78.1
11	Nooralsana	2012	Fasa	36.4±12.1	235	235	470	77.5
12	Nasiriani	2008	Yazd	38	53	47	100	67.5
13	Golafrooz	2001	Sabzevar	1-90	NA*	NA*	193	95.3
14	Kianmehr	2008	Tehran	NA*	NA*	NA*	638	62
15	Seydi	2006	Ghom	NA*	NA*	NA*	180	71.14
16	Sheykhi	2000	Ghazvin	15-24	42	29	71	59
17	Shojaii	2008	Kerman	31.3±14.3	257	133	390	75.3
18	Soleimanpour1	2008	Tabriz	NA*	175	128	303	62
19	Saadati	2004	Mashhad	NA*	406	326	732	61.7
20	Soleimanpour2	2011	Tabriz	NA*	296	204	500	63.2
21	Jalili	2006	Tehran	42.5	NA*	NA*	317	56.61
22	Kazemifard	2011	Jahrom	NA*	NA*	NA*	526	80.2
23	Abrakht	2011	Booshehr	NA*	NA*	NA*	483	35
24	Janati	2011	Tabriz	NA*	NA*	NA*	178	76
Total	Pooled estimate	-	-	-	2215	2385	11168	68.9(57.2-80.7)
	Heterogeneity test	Chi- square(Q)	-	-	-	-	-	7272(P<0.001)
		I-Square	-	-	-	-	-	99.7%(P<0.001)
Total(after removal of the outlier data)	Pooled estimate	-	-	-	-	-	-	70.9(66.1-75.6)
	Heterogeneity test	Chi- square(Q)	-	-	-	-	-	314.7(P<0.001)
		I-Square	-	-	-	-	-	95.2%(P<0.001)

* : Not Available.

**Table 2:** Checklist to assess the primary studies

No	Questions	Score	

		yes=1	No=0
1	Are the research questions or objectives clearly stated?		
2	Is the study context clearly described?		
3	is the sample size stated?		
4	Is the calculation of sample size clearly described (is the sample size appropriate According to the research question and variables?)		
5	Is the sampling method clearly described?		
6	Is the sampling strategy appropriate for the research question?		
7	Is the method of data collection clearly described?		
8	Is the data collection method appropriate to the research question?		
9	Is the method of analysis clearly described?		
10	Are the research results clearly stated?		
11	Is the analysis appropriate for the research question?		
12	Are the claims made supported by sufficient evidence?		

According to this checklist, maximum score is 12 (Moosazadeh, Nekoei-moghadam, Emrani, & Amiresmaili, 2014). Finally, the primary articles which obtained score 8 and more, were selected and the related information was extracted and analyzed.

### 2.4 Studies Inclusion Criteria

All Persian and English studies which, attained the predetermined score of eight and which determined the rate of hospital emergency patient satisfaction of Iran were included.

### 2.5 Studies Exclusion Criteria

After reviewing and examining articles or abstract of articles and after recognizing disagreements, following studies were excluded:

A) Studies that did not report overall satisfaction of patients from the emergency departments, B) studies which sample size was not specified, C) proceedings of congresses and conferences which full texts were not available.

### 2.6 Data Extraction

Data was extracted by researchers in terms of title, first author, publication year, total sample size, sample size by gender, research method, research place, total rate of satisfaction and rate of satisfaction in terms of gender. Data was entered into Excel spreadsheet.

### 2.7 Analysis

Data was entered into Stata software for analysis. Standard error of patient’s satisfaction level in each study was calculated using the binomial distribution formula. Finally, the heterogeneity index was determined among studies using the Cochran test (Q) were determined. Based on the heterogeneity results with meta-command (meta) in the meta-analysis, random effect model was used to estimate total satisfaction level of patients with the emergency rooms in Iran. In addition, to minimize the random distribution between point estimates of the studies, the findings of all studies were adjusted using Bayesian analysis. Finally, the effects of variables which had been determined as possible sources of heterogeneity were examined in stata 11 software using Meta-regression method. Spot estimates of patients’ satisfaction level with emergency rooms were calculated at forest plots (confidence interval=95%). In this graph, size of the square represents weight of each study and lines at both sides of the square show confidence interval of 95%.

## 3. Results

**Figure 1:**
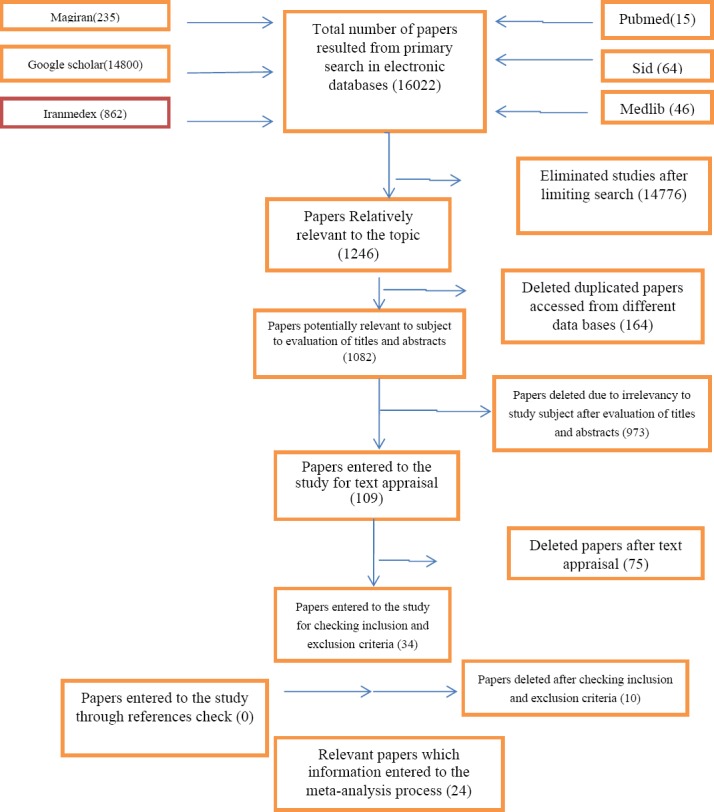
Papers search and review flowchart

Using the related keywords with the maximum sensitivity, 16022 articles were found in national and international electronic databases; limiting the keywords and increasing search properties, 1246 articles were selected. Of them, 146 cases were excluded due to duplication. Titles and abstracts of 1082 articles were assessed, and 973 unrelated cases were excluded. Then, full texts of 109 articles were reviewed and 75 unrelated cases were omitted again. 34 related articles were evaluated with a checklist of quality control and inclusion and exclusion criteria. Of them, 10 articles were excluded due to some reasons including failure to achieve minimum score of quality assessment, unclear sample size and unclear percentage of satisfaction with emergency rooms of hospitals. Finally, 24 articles were entered into the meta-analysis. The references of articles were also reviewed in order to increase the sensitivity of the search, but no new article was found (Figure 1, Table 1).

All initial studies which were entered into the meta-analysis were of descriptive-analytic type. Random sampling method (simple or systematic) had been used in 15 studies; stratified sampling method had been used in 2 studies, simple method in one case; sequential sampling method in one study; and 5 studies had unclear sampling method. A checklist was used to collect data in all studies. Its validity had been reported in 18 studies. It should be noted here that content of the checklist used in the initial studies was different. The aim of all studies was to determine patients’ satisfaction level with the way services were presented in emergency centers of hospitals.

Total number of samples examined in initial studies which were entered into the meta-analysis was 11,168 patients. Only 12 studies reported their sample size by sex (2215 males and 2385 females). The highest sample size was related to a study carried out by Zahmatkesh in Golestan Province (N=2,400) and the lowest one was related to a study by Peyrovi in Tehran (N=20). The lowest and highest satisfaction level was 24 (Zahmatkesh, Hajimoradloo, Kazemi Malekmahmoodi, & Khoddam, 2010) and 98.4% (Sarchami, & Sheikhi, 2006) respectively.

**Figure 2:**
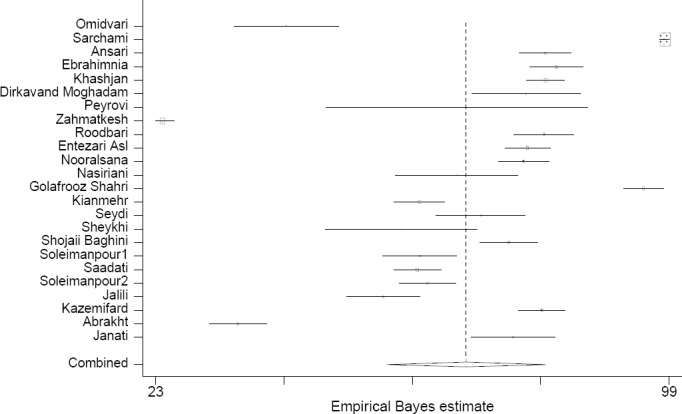
Distribution of the adjusted percent of patient satisfaction in each study and overall; This chart shows that the range of patient satisfaction percent in each study is 24.04-98.39% before removing outlier data, pooled estimate: 68.9% (57.2-80.7), I2=99.7%

**Figure 3:**
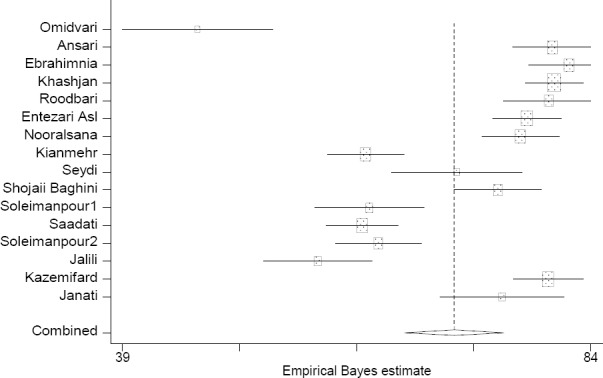
Distribution of the adjusted percent of patient satisfaction in each study and overall; This chart shows that the range of patient satisfaction percent in each study is 46.2-81.9% after removing outlier data, pooled estimate: 70.9% (66.1-75.6), I2=95.2%

This meta-analysis revealed that patients’ total satisfaction with emergency sections of hospitals in Iran was 68.9% (57.2-80.7) based on the random effect model (according to presence of heterogeneity in initial studies) (Figure 2). In order to study the effects of initial studies on heterogeneity, sensitivity analysis was also done. Although heterogeneity level decreased drastically after recognizing and excluding some studies, it still existed. After studies affecting heterogeneity were excluded, total satisfaction with emergency departments was 70.9 (66.1-75.6) (Figure 3). Using meta-regression, publication year was examined as one of the possible sources of heterogeneity; it had no significant effect on heterogeneity of initial studies (P=0.2, B= -1.3).

## 4. Discussion

This meta-analysis determined patients’ total satisfaction with the way services were presented and with the quality of services in hospital emergency rooms in Iran. According to the results of this meta-analysis, patients’ satisfaction level with services presented at hospital emergency rooms was 70.9 with regard to the heterogeneity of the studies and based on the random effect model.

In a study conducted by Abolhasani & Tavakol in 1994 in Hamedan, satisfaction level of patients’ relatives with services presented at emergency rooms was 47.1% (Abolhassani & Tavakol, 1994). Moreover, it has been shown in studies conducted in western countries that culture of the society and interaction with patients affect patients’ satisfaction (Taylor & Benger, 2004). In a study by Shaikhi and Javadi, patients’ satisfaction level with admission at the emergency rooms was 94.4% (Shaikhi & Javadi, 2005). In another study conducted by Ansari et al in 2004 in Iran University of Medical Science, total satisfaction level with emergency services and emergency admission services was 80.7 and 86.5% respectively. In his study, Ansari stated that high treatment costs at emergency centers, buying equipment, waiting at emergency rooms, not using curtains or partitions and bad conditions of emergency rooms were the most important reasons of dissatisfaction (Ansari, Ebadi, & Molasadeghi, 2005). In his study, Shojaie Baghini et al reported that total satisfaction level with emergency rooms was 75.3% (Shojaee Baghini, Nakhei, Bahrampour, Namazian, & Mehrabian, 2008). High satisfaction level in the above study may be due to the differences in people’s culture, their demands, and differences in number of people referring to emergency rooms (Vieth & Rhodes, 2006) or differences in quality of the presented services (Cassidy–Smith, Baumann, & Boudreaux, 2007).

In a study by Shojaie Baghini, a significant relationship was observed between age, referral reason and satisfaction level (P=0.008); as age and health status improve, satisfaction level increases. Also, there was a significant relationship between number of referrals to emergency departments (P=0.01) and hospitalization (P=0.01) and satisfaction of patients’ relatives (Shojaee Baghini, Nakhei, Bahrampour, Namazian, & Mehrabian, 2008). In studies conducted in developed countries, age is a factor influencing clients’ satisfaction with services presented in emergency rooms (Taylor, & Benger, 2004). In a study which examined the quality of care in 13 emergency centers in Canada, satisfaction level was 73.1% (Hutchison et al., 2003).

Results of similar studies show that satisfaction of patients hospitalized in emergency centers of Ahvaz hospitals was 86% (Taheri, Fereydounimoghadam, Cheraghian, & Khazani, 2010); and it was 98.4% in treatment-educational centers affiliated to Qazvin University of Medical Science and Health-Treatment Services (Sarchami & Shakhi, 2003). This difference in satisfaction level may be due to difference in subjects, types of questions and scales as well as ignoring private hospitals and shortage of number and physical space of emergency centers.

In their study, Thomas et al reported that the effect of equipment and tools of emergency centers and presence of necessary drugs were 96.0% and 85% respectively (Thomas et al., 1995). This difference in satisfaction level may be due to some factors including cultural differences and familiarity of patients and their relatives with their rights. Karimollahi and Mazaheri reported that access to emergency rooms, cleanness, temperature, ventilation and conditions of bathrooms were the factors affecting patients’ satisfaction (Karimollahi & Mazaheri, 2000).

In a study by Sarchemi, satisfaction level with emergency centers (98.4) was significantly different in terms of hospital, sex and age of patients, number of referrals to emergency centers, insurance type and status and patient’s fate (Sarchami & Shakhi, 2003).

Like results of some studies done in other countries, results of a study carried out by Omidvari reflected the relationship between age, gender, educational level and length of stay and patients’ satisfaction with the care they had received (Omidvari et al., 2008).

Using a structured search of all available initial studies, the present meta-analysis tried to depict a clear image of patients’ satisfaction with emergency rooms of hospitals in Iran by reviewing quality of initial studies, excluding unrelated studies and increasing power of the studies by employing high sample volumes. Thus, this evaluation can be used by policy makers and programmers.

One of the limitations of the present study which was related to the initial studies was scattered variables and different contents of information collection tools; it affected total satisfaction level reported in every study and was considered a possible source of heterogeneity. Another possible limitation was lack of access to certain documents because they were not published. It should be noted that although sex could be a factor affecting satisfaction level, evaluating this index was impossible because satisfaction level had not been reported in terms of sex. Another limitation was that initial studies focused in a limited number of provinces (14 provinces), and it was not taken into account in many provinces despite the importance of emergency centers.

## 5. Conclusion

This meta-analysis showed that patients’ satisfaction with the performance of hospitals and the way services were presented in emergency centers in hospitals of Iran was desirable. Concerning multifactorial nature of patients’ satisfaction, it is necessary to take this matter into regular and routine consideration. It is suggested that future studies will focus on all provinces and that patients’ satisfaction will be determined and compared in both private and governmental hospitals.
